# pSTAT3 Levels Have Divergent Expression Patterns and Associations with Survival in Squamous Cell Carcinoma and Adenocarcinoma of the Oesophagus

**DOI:** 10.3390/ijms19061720

**Published:** 2018-06-10

**Authors:** Katie E. O’ Sullivan, Adriana J. Michielsen, Esther O’ Regan, Mary C. Cathcart, Gillian Moore, Eamon Breen, Ricardo Segurado, John V. Reynolds, Joanne Lysaght, Jacintha O’ Sullivan

**Affiliations:** 1 Trinity Translational Medicine Institute, Department of Surgery, Trinity College Dublin, St James’s Hospital, Dublin 8, Ireland; kaosulli@me.com (K.E.O.S.); jannie.michielsen@gmail.com (A.J.M.); maryclarecathcart@gmail.com (M.C.C.); mooregy@tcd.ie (G.M.); breenea@tcd.ie (E.B.); reynoljv@tcd.ie (J.V.R.); jlysaght@tcd.ie (J.L.); 2 Department of Histopathology, St James’s Hospital, Dublin 8, Ireland; emoregan@gmail.com; 3 Centre for Support and Training in Analysis and Research (CSTAR), University College Dublin, Dublin 4, Ireland; ricardo.segurado@ucd.ie

**Keywords:** STAT3, oesophagus, cancer, squamous cell carcinoma, adenocarcinoma

## Abstract

Signal transducers and activator of transcription (STAT)-3 is activated in cancers, where it promotes growth, inflammation, angiogenesis, and inhibits apoptosis. Tissue microarrays were generated using tissues from 154 patients, with oesophageal adenocarcinoma (OAC) (*n* = 116) or squamous cell carcinoma (SCC) (*n* = 38) tumours. The tissues were stained for pSTAT3 and IL-6R using immunohistochemistry. The OE33 (OAC) and OE21 (SCC) cell lines were treated with the STAT3 inhibitor, STATTIC. The Univariate cox regression analysis revealed that a positive pSTAT3 in SCC was adversely associated with survival (Hazard ratio (HR) 6.382, 95% CI 1.266–32.184), while a protective effect was demonstrated with the higher pSTAT3 levels in OAC epithelium (HR 0.74, 95% CI 0.574–0.953). The IL-6R intensity levels were higher in the SCC tumours compared with the OAC tumours for the core and leading edge tumour tissue. The pSTAT3 levels correlated positively with the IL-6R levels in both the OAC and SCC. The treatment of OE21 and OE33 cells with the STAT3 inhibitor STATTIC in vitro resulted in decreased survival, proliferation, migration, and increased apoptosis. The pSTAT3 expression was associated with adverse survival in SCC, but not in the OAC patients. The inhibition of STAT3 in both of the tumour subtypes resulted in alterations in the survival, proliferation, migration, and apoptosis, suggesting a potential role for therapeutically targeting STAT3.

## 1. Introduction

Oesophageal cancer is the eighth most common malignancy worldwide and the sixth leading cause of cancer-related deaths [1]. Oesophageal cancer is an aggressive disease with a poor prognosis and the 5-year survival rate for patients with distant metastasis is only 3% [2]. The mainstay of treatment is an oesophagectomy, with or without neoadjuvant chemoradiotherapy or neoadjuvant, and adjuvant chemotherapy. The molecular profiling of tumours allowing tailored therapy in other malignancies is advancing, however this approach is at an early stage in cancers of the oesophagus [3].

Signal transducer and activator of transcription (STAT)-3 is activated in many cancers, where it promotes growth, inflammation, angiogenesis, and inhibits apoptosis [4]. STAT proteins play dual roles by transmitting the information that is received from a wide variety of extracellular polypeptide signals via their cognate cell surface receptors, providing a mechanism for the regulation of gene transcription [5]. Binding leads to the activation of receptor-associated janus kinase (JAK) kinases and JAK-mediated phosphorylation of the cytoplasmic receptor domains [6]. The phosphorylation of receptors results in STAT protein recruitment, phosphorylation, and dimerisation. This results in nuclear translocation and transcriptional control via DNA binding, to target the gene regulatory sequences [6]. Gene targets include cyclin B1, cyclin D1/2, cdc2, and c-myc, whose transcriptional upregulation results in progression through the G1/S phase of the cell cycle. Another group of STAT3 targets the genes that induce VEGF and HIF1-α induced angiogenesis. This results in tumour revascularization [7], while MMP2 and MMP9 facilitate with the tumour metastasis [7]. The cytokines implicated in the activation of STAT3 signaling include oncostatin M (OSM), interleukin-6 (IL-6), and Leptin [8,9,10].

Persistent activation of phosphor-STAT3 (pSTAT3) is thought to contribute to apoptotic resistance in oesophageal squamous cell carcinoma (SCC) cells [11]. In SCC, the pSTAT3 expression correlates positively with the pathological TNM stage and metastasis, and the increased pSTAT3 expression is correlated with a shorter overall survival [12]. Furthermore, the constitutive activation of pSTAT3 in SCC results in upregulation of the total STAT3, VEFG, and Bcl-2 mRNA expression [13]. Furthermore, the electrophoretic mobility gel shift (EMSA) studies show the high DNA-binding capabilities of pSTAT3 in SCC [13]. The role of pSTAT3 activation in adenocarcinoma is less well understood. There is a significant association between obesity and oesophageal adenocarcinoma. Therefore, pSTAT3 activation by inflammatory adipokines could contribute to obesity-driven oesophageal adenocarcinoma [14]. There is evidence that suggests the distinct signaling activity in the two main histological subtypes, with altered functional cellular responses following STAT3 inhibition in oesophageal adenocarcinoma (OAC) and SCC cells [15]. Furthermore, an investigation of the transcriptome analysis of the STAT3 knockdown revealed that the reduced STAT3 was associated with the downregulation of the cell cycle genes in both cell types, but, interestingly, there were divergent alterations in genes promoting cell migration. The reasons for the divergent signaling patterns between the subtypes remains unclear. Manipulation of STAT3 as a therapeutic target has been explored and a number of small molecule STAT3 inhibitors exist. It has been shown that the small molecule inhibitor, STATTIC, is cell permeable and can block the phosphorylation of tyrosine in the SH2 domain of STAT3 and inhibit the binding of dimerised STAT3 to its specific DNA response element [16]. We chose this compound to inhibit STAT3 and have examined the functional cellular effects. Additionally, we undertook a study to examine the two key signaling components, namely, IL-6R and pSTAT3 in SCC and OAC tumours, respectively. This study examines the survival associations of pSTAT3 signalling in a large cohort of both OAC and SCC patients, in addition to the functional cellular effects of STAT3 inhibition in both subtypes, in order to demonstrate divergent STAT3 signalling patterns in the main histological subtypes of oesophageal cancer.

## 2. Results

### 2.1. Divergent Associations of pSTAT3 and Survival in OAC versus SCC

**Figure 1:**
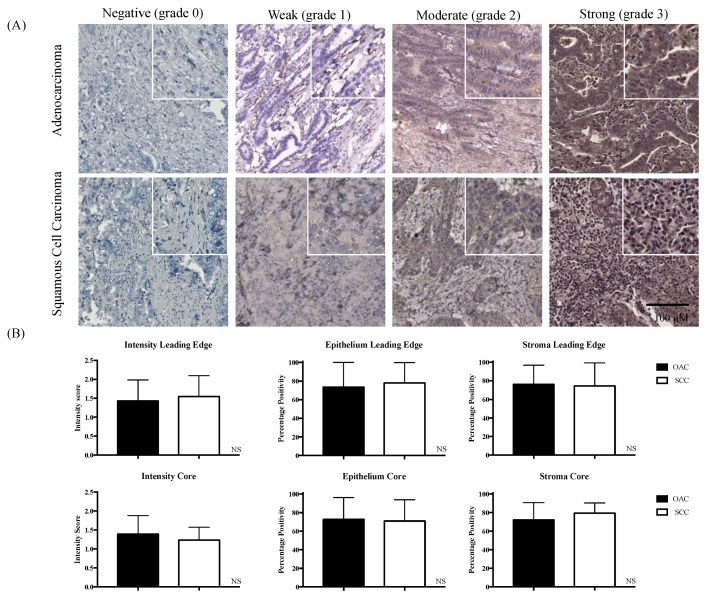
Tissue microarray grading and p-signal transducer and activator of transcription (STAT)3 values in oesophageal adenocarcinoma (OAC) and squamous cell carcinoma (SCC). (**A**) Paraffin-embedded tissue microarrays of oesophageal adenocarcinoma and squamous cell carcinoma were stained using immunohistochemistry for pSTAT3 expression. Intensity was scored from 0–3, where grade 0 was negative, grade 1 was weak, grade 2 moderate, and grade 3 was strong staining. In addition, the percentage positivity in the epithelium and stroma was scored between 0, 10, 25, 50, 75, 90, or 100% positivity. Magnification was 10×. Further magnification of images is shown in the upper right of each example. (**B**) The mean values of intensity and positivity of pSTAT3 in OAC and SCC leading edge and core specimens. Statistical analysis was carried out using Mann–Whitney U testing.

Resected tumour specimens from patients with OAC and SCC were evaluated using immunohistochemistry (IHC) for pSTAT3. Figure 1A shows representative images of weak and strong pSTAT3 staining in OAC and SCC tissue. The mean intensity levels and percent positivity (epithelium and stroma) in the core and leading edge tissues were similar in OAC and SCC tumours (Figure 1B). However, on the univariate cox regression analysis, the positive tumour pSTAT3 stromal staining in core SCC samples was associated with death (HR 6.382, 95% CI 1.266–32.184). The core SCC finding did have a time-varying effect included, indicating that pSTAT3 was at high-risk in an earlier follow-up, but more ambiguous thereafter.

Conversely, a protective effect was demonstrated with the higher pSTAT3 levels in OAC epithelium in the leading edge samples (HR 0.74, 95% CI 0.574–0.953) (Table 1). The Kaplan–Meier curves of the association of pSTAT3 and leading edge OAC and core SCC are shown in Supplemental Figures S1 and S2 Regarding the pSTAT3 expression in the epithelium and stroma of SCC, when examining the percentage positivity in the epithelium vs. the stroma of the leading edge samples, there was no significant difference (*p* = 0.67). Regarding percentage positivity in the epithelium vs. the stroma in the core samples, there was no significant difference (*p* = 0.06). When combining the leading edge and the core scores, there was no significant difference (*p* = 0.12). Regarding the pSTAT3 expression in epithelium and stroma of OAC, for the leading edge there was no significant difference (*p* = 0.054), for the core there was no significant difference (*p* = 0.42), also and when combining the leading edge and the core there was no significant difference (*p* = 0.4).

When examining the percentage positivity epithelium multiplied by percentage positivity stroma, the following results were obtained. For the leading edge, only comparing the scores of OAC vs. SCC, there was no significant difference (*p* = 0.58); for the core scores, comparing OAC and SCC, there was no significant difference (*p* = 0.66); and when combining the leading edge and the core scores, there was no significant difference (*p* = 0.9).

### 2.2. IL-6R Expression in OAC and SCC

**Figure 2:**
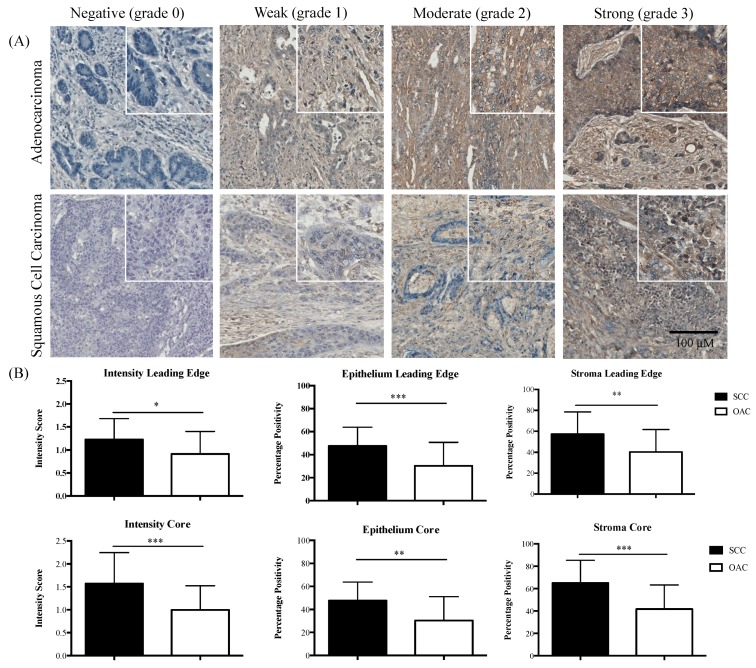
Tissue microarray grading and IL-6R values in OAC and SCC. (**A**) Paraffin-embedded tissue microarrays of oesophageal adenocarcinoma and squamous cell carcinoma were stained using immunohistochemistry for the IL-6R expression. The intensity was scored from 0–3, where 0 was negative, grade 1 was weak, grade 2 was moderate, and grade 3 was strong staining. In addition, the percentage positivity in the epithelium and stroma were attributed a score of 0, 10, 25, 50, 75, 90, or 100%. The magnification was 10×. Further magnification of images is shown in the upper right of each example. (**B**) The mean values of intensity and positivity of pSTAT3 in the OAC and SCC leading edge and core specimens. Statistical analysis using Mann–Whitney U testing. Differences of *p* < 0.05 (*), *p* < 0.01 (**), and *p* < 0.001 (***) were considered statistically significant.

**Figure 3:**
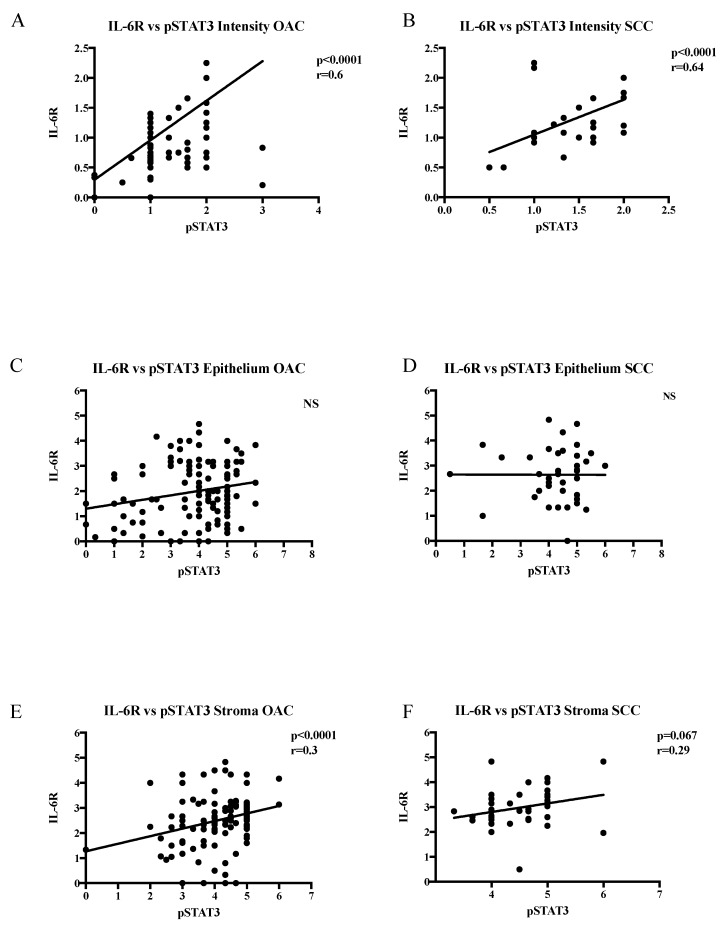
(**A**–**F**) Correlation between pSTAT3 and IL-6R immunohistochemistry scores in OAC and SCC tissue microarray (TMA) specimens. (**A**) Intensity scoring OAC; (**B**) intensity scoring SCC; (**C**) epithelial scoring OAC; (**D**) epithelial scoring SCC; (**E**) stromal scoring OAC; and (**F**) Stromal scoring. Spearman’s correlation testing was used. Differences of *p* < 0.05 (*), *p* < 0.01 (**), and *p* < 0.001 (***) were considered statistically significant.

The representative images of the weak and strong IL6R expressions are shown in Figure 2A. The IL-6R intensity levels were significantly higher in the SCC tumours compared with the OAC tumours for the core and leading edge tumour tissue (*p* = 0.01, *p* < 0.0001, respectively). The expression levels of IL6R in the epithelial and stromal compartments in the SCC tumours was significantly higher compared with the OAC tumours (for the core and leading edge tissue, all *p* values < 0.001) (Figure 2B). Using Spearman’s correlation testing, the pSTAT3 levels correlated positively with the IL-6R levels in both OAC and SCC, suggesting an important role for IL-6 in STAT3 activation in both of the subtypes (Figure 3). Notably, the association between the IL-6R expressions was examined and a high IL-6R was associated with an adverse survival in the stroma of the OAC core specimens (Supplementary Table S1). Furthermore, the association between the pSTAT3 and IL-6R expressions and tumour differentiation, ratio of positive nodes, pathological T stage, and pathological N stage were examined and are reported (Supplementary Tables S2 and S3). All of the results were non-significant, apart from the correlation between the percentage positivity in the epithelium and the pathological N stage in the leading edge samples of OAC.

### 2.3. Functional Effects of STAT3 Inhibition on SCC and OAC In Vitro

#### 2.3.1. Cell Viability

**Figure 4:**
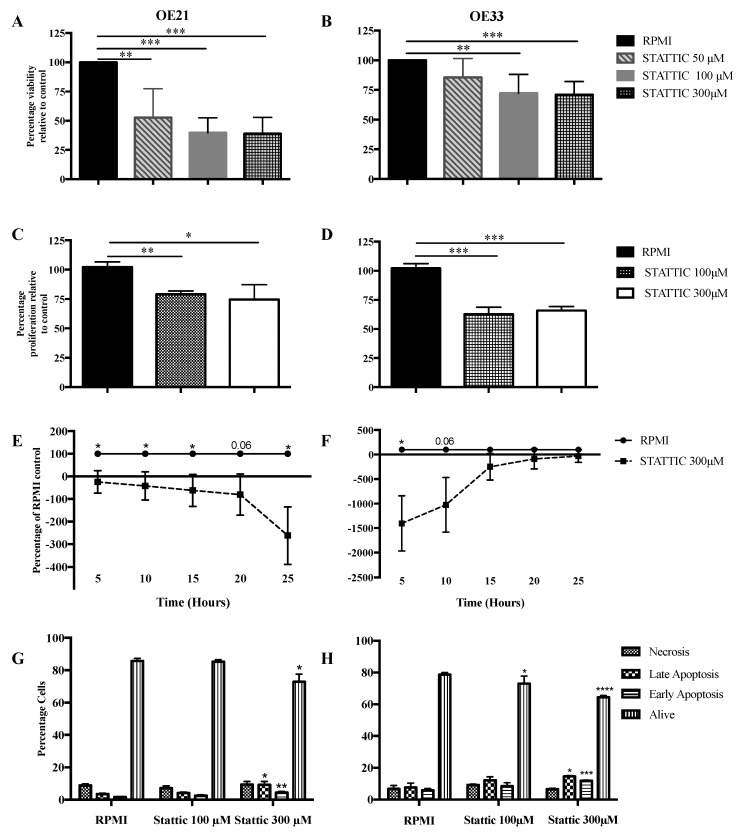
Functional effects of STAT3 inhibition in SCC and OAC. (**A**) The survival of OE21 and (**B**) OE33 cells was measured by a 3-(4,5-dimethylthiszol-2-yl)-2,5-diphenyltetrazolium bromide (MTT) assay after 24 h treatment with STAT3 inhibitor STATTIC, 50 μM, 100 μM, and 300 μM, relative to the RPMI control. (**C**) The effect of STATTIC on the cell proliferation of OE21 and (**D**) OE33 was assessed using a Bromodeoxyuridine (BRDu) assay, following treatment with STATTIC, 50, 100 and 300 μM, relative to the RPMI control. Bars denote the mean standard deviation (SD). (**E**) Cellular migration assays were performed on the XCelligence platform. Cell migration of OE21 and (**F**) OE33 was assessed over time, following treatment with STATTIC 300 μM, relative to the RPMI alone. The (**G**) OE21 and (**H**) OE33 cells were treated with STATTIC, 100 μM and 300 μM for 24 h, relative to the RPMI control. The cells were stained with Annexin-V and propodium iodide, and an analysis was performed using flow cytometry. *p* values were estimated using the student’s *t*-test. Differences of *p* < 0.05 (*), *p* < 0.01 (**), *p* < 0.001 (***) and *p* < 0.0001 (****) were considered statistically significant. *N* = 3.

A series of treatments of OE33 and OE21, using STATTIC at a range of concentrations, were performed to determine the optimum dose for this cell line (Supplementary Figure S3). Following the treatment of the OE21 (SCC) cells with the STAT3 inhibitor STATTIC, there was a significant decrease in the cell viability using 50 μM (*p* = 0.0027), 100 μM (*p* < 0.0001), and 300 μM (*p* < 0.0001) concentrations, relative to Roswell Park Memorial Institute (RPMI) medium as the control (Figure 4A). Following the treatment of the OE33 (OAC) cells with STATTIC, there was a significant decrease in the cell viability using 100 μM (*p* = 0.0045) and 300 μM (*p* = 0.0004) concentrations, relative to RPMI medium as the control (Figure 4B).

#### 2.3.2. Cell Proliferation

Following the treatment of the OE21 cells with STATTIC, there was a significant decrease in the proliferation, relative to the control, using 100 μM (*p* = 0.0015) and 300 μM (*p* = 0.023) concentrations (Figure 4C). The treatment of OE33 cells with STATTIC also resulted in a decrease in proliferation, using 100 μM (*p* = 0.0007) and 300 μM (*p* = 0.0003) concentrations (Figure 4D).

#### 2.3.3. Cell Migration

The treatment of OE21 cells with STATTIC, 300 μM, resulted in a significant decrease in the cell migration, relative to the RPMI medium control at 5, 10, 15, and 25 h time points (*p* = 0.02, *p* = 0.04, *p* = 0.04, and *p* = 0.01, respectively) (Figure 4E). In contrast, the treatment of OE33 cells with STATTIC, 300 μM, resulted in a significant decrease in the cell migration at 5 h only (*p* = 0.02) (Figure 4F).

#### 2.3.4. Apoptosis

The treatment of the OE21 cells with STATTIC, 100 μM, did not result in any significant alterations in the early or late apoptosis or necrosis, relative to the RPMI control. Treatment with STATTIC, 300 μM, resulted in a significant increase in the early (*p* = 0.0049) and late apoptosis (*p* = 0.04), and a significant decrease in the live cells (*p* = 0.03), relative to the RPMI control (Figure 4G). The OE33 cells that were treated with STATTIC, 100 μM, demonstrated a trend for increased early apoptosis, however, this did not reach significance (*p* = 0.06). There was no difference in the late apoptosis (*p* = 0.09) or necrosis (*p* = 0.2), relative to the RPMI control. There was, however, a significant decrease in the live cells (*p* = 0.04), relative to the RPMI. The treatment of OE33 cells with 300 μM STATTIC resulted in a significant increase in early apoptosis (*p* = 0.0004) and late apoptosis (*p* = 0.02). Additionally, there was a significant decrease in the live cells (*p* < 0.0001), relative to the RPMI control (Figure 4H).

## 3. Discussion

Signal transducer and activator of transcription (STAT) proteins have a critical role in oncogenic signaling in a number of malignancies, and understanding their profile and activation may provide a beneficial therapeutic target in oesophageal cancer [17,18,19]. The pathogenesis of oesophageal SCC and OAC is distinct. Understanding the contribution and activation patterns of the distinct signaling pathways in each histological subtype is of specific interest for the development of targeted therapies, to be used in combination with existing chemoradiotherapy/surgery regimes and with other receptor tyrosine kinase inhibitors.

In this study, we demonstrated that the elevated levels of pSTAT3 were associated with poorer survival in oesophageal SCC, with the inverse having been identified for pSTAT3 in the OAC tumours. A similar association was recently published in the context of chronic lymphocytic leukaemia and, although a large body of literature linked STAT3 with adverse survival overall, this finding, in addition to the divergent association seen with SCC, was reflective of the complexity of STAT3 signalling in tumours [20]. As STAT3 signaling is exquisitely regulated within cells, it was therefore possible that the OAC tumours over the course of their progression developed mechanisms whereby they could suppress the effect of STAT3 activation, such as through the upregulation of proteins that negatively regulated the STAT signaling. The finding of a protective effect in OAC was, however a novel one. Regarding prior studies, a systematic review and meta-analysis by Li et al. had examined the prognostic significance of the pSTAT3 expression in cancers of the digestive system [21]. The overall findings suggested that elevated pSTAT3 levels were a strong predictor of overall inferior and disease free survival. Chen et al. had examined the role of acylglycerol kinase (AGK) in oesophageal SCC and had found that this lipid kinase was over expressed in SCC and was correlated with overall poorer survival. Furthermore, the authors determined that it bound directly with the JH2 domain of the JAK2 to block the JH3 mediated inhibition of JAK2, which resulted in a constitutive activation of JAK2/STAT3 in SCC, both in vitro and in vivo, thus hypothesising that AGK might be an interesting prognostic factor, with significance in terms of determining the optimum therapeutic strategy for patients with SCC of the oesophagus [22].

Our findings are highly suggestive of very divergent STAT3 activation and action between OAC and SCC. A study by Timme et al. examined the expression of the total STAT3 and pSTAT3 in OAC and SCC, and found differential expression across the subtypes, with more frequent nuclear pSTAT3 positivity in the nucleus of the SCC samples [15]. Indeed, a number of other studies associated an adverse prognosis with increased STAT3 in oesophageal SCC [12,23]. For the first time, our study interestingly identified a protective association between the STAT3 expression and survival in OAC. Mesteri et al., observed differential mesenchymal-epithelial transition factor (cMet) levels, comparing the OAC and SCC tumour tissue. In the OAC, but not the SCC, the cMet expression correlated with EGFR, pSTAT3 expression, and lymphovascular invasion of tumour cells [24]. Additionally, the cMet overexpression was associated with shorter disease-free survival as well as disease specific and overall survival of OAC, but not SCC patients [24]. One potential reason for the differences that were seen was the issue of obesity driven pSTAT3 activation. As described by Yu et al., there was evidence to suggest that the activation of the JAK2-STAT3 pathway in epithelial cells could promote the development of obesity-associated cancer through the activation of inflammatory immune responses [25].

Furthermore, STAT3 inhibition could increase radiosensitivity as well as reduce cell growth and angiogenesis in SCC [23,26]. In a study of human oesophageal squamous cell carcinoma by Liu et al., the inhibition of p-JAK2 and p-STAT3 expression using a selective COX-2 inhibitor Nimesulide, resulted in a growth inhibition in Eca-109 squamous cell carcinoma cells [27].

Compared with SCC, less is known about the independent prognostic significance of pSTAT3 activation in oesophageal adenocarcinoma. Schoppmann et al., examined tumours from 324 patients with OAC and SCC and 45 patients with precursor lesions, and demonstrated that STAT3 played a role in the development of oesophageal adenocarcinoma, however, only the combination with Her-2 expression status was associated with an adverse survival outcome [28]. Our analysis demonstrated a significantly higher level of IL-6R expression in SCC compared with OAC, suggesting a more prominent role for activation of the signaling pathway by IL-6 in SCC. Whilst this was one activating cytokine, in this context, it was acknowledged that there were other cytokines activating this pathway. This was supported by Chen et al., who identified the expression IL-6 to be strongly associated with a poor prognosis in patients with SCC [29]. Furthermore, similar to our data that demonstrated a correlation between IL-6 and pSTAT3, they revealed that the IL-6 neutralising antibody had a similar effect on the EMT-related protein levels as the STAT3, siRNA, or JAK inhibitor, suggesting that the altered STAT3 activation in oesophageal SCC was in part because of the IL-6/STAT3 activation. 

In our study, we demonstrated significant reductions in cell viability and proliferation with increased apoptosis in both OAC and SCC cell lines, following treatment with the STAT3 inhibitor, STATTIC. Examining SCC in isolation, Cao et al., recently published data supporting this finding. Following the treatment of KYSE150 and KYSE450 cell lines with STAT3-PLK-1-AKT signalling blocker plumbagin, there was a reduction in the proliferation and an induction of apoptosis in the SCC cells via the abrogation of STAT3-PLK-1-AKT signalling [30]. Interestingly, the authors also found that constitutively activated mutant STAT3C reinstated the plumbagin blockade of signalling [30].

We demonstrated a decrease in migration in both of the cell lines initially, but recovery in a migratory capacity within the OAC cells, while it remained reduced in SCC cells. The reasons for this were unclear, however, we hypothesized that the migratory inhibition in OE33 cells may be transient. The migratory inhibition in SCC was reported previously through the inhibition of pSTAT3 in a number of studies, which were thought to occur via the regulation of EMT markers and matrix metalloproteinases [31,32,33]. Less, however, was known regarding the impact of STAT3 inhibition on cell migration in OAC. Timme et al., examined the functional effects of STAT3 knockdown in both OAC and SCC, and demonstrated reduced cell migration in both of the cancer subtypes. Whilst their migration assay was performed on the same platform, the timeframe of their analysis was significantly different, with their study examining timepoints 96–144 h and ours examining 0–25 h, as was their method of STAT3 knockdown [15]. Further studies would be required to determine the differential effect of STAT3 on OAC and SCC cell migration.

## 4. Materials and Methods

### 4.1. Patient Recruitment

All of the patients undergoing a surgical resection for oesophageal cancer at St. James’s Hospital were invited to partake. Only the patients with no history of cancer prior to their diagnosis of oesophageal cancer were included. Informed consent was obtained following the ethical approval from the AMNCH/St. James’s Hospital Institutional Ethics Committee. Ethics ref number 04113/10804, granted November 2004. The tumour samples were obtained during surgical resection.

### 4.2. Tissue Microarray Construction

Tissue microarrays (TMAs) were created from paraffin-embedded OAC and SCC resection specimens that were collected between July 1998 and December 2009. A pathologist marked the areas containing viable tumour cells. Cores were taken from tumour-containing areas and arrayed in a paraffin block. Where possible, the invasive edge of the tumour was marked and included. For a total of 154 patients (*n* = 116 OAC, *n* = 38 SCC) we had core specimens and for 102 patients we had leading edge specimens (*n* = 76 OAC, *n* = 24 SCC). A full breakdown of the details is shown in Table 2. 

The data pertaining to the patient demographics, clinicopathological stage, and outcomes were obtained from the oesophageal cancer database. The tumour, node, and metastasis (TNM) descriptors and the staging classification that were used were those that were defined by the seventh edition of the American Joint Committee on Cancer staging manual [34].

### 4.3. Immunohistochemistry

Immunohistochemical (IHC) staining for pSTAT3 and IL-6R was optimized by staining the full-face sections of the resected oesophageal cancer paraffin-embedded blocks. The sections were reviewed to ensure tissue integrity, staining specificity, and optimum antibody concentration. TMA sections of 4 μm were stained with anti-IL6R (Abcam (Cambridge, MA, USA) ab128008, isotype: rabbit polyclonal, dilution 1:200) and anti-pSTAT3 (Cell Signalling (Brennan and Company, Co. Dublin, Ireland), Tyr705, D3A7 isotype: rabbit monoclonal, dilution 1:400). The sections were counter-stained with haematoxylin (Sigma, St. Louis, MO, USA). The TMAs contained tissue from patients with oesophageal cancer that were diagnosed between 1998 and 2009. Of these, 116 had a diagnosis of oesophageal adenocarcinoma and 37 had a diagnosis of squamous cell carcinoma. The grading was performed by multiple observers (KOS, JM, EOR), one of which was a consultant histopathologist (EOR).

Scoring was on the basis of intensity (0–3), where zero was negative, one was weak, two was moderate, and three represented strong staining. The quantity of positive staining within the core was scored 0, 10, 25, 50, 75, 90, or 100%. The average overall score for each of the three cores per patient was calculated and the mean score for each patient was attained. A biostatistician from the Centre for Support and Training in Analysis and Research (CSTAR) was consulted with respect to the statistical analysis of the dataset. The Cox Proportional Hazard regression was used to assess the influence of pSTAT3 and tumour characteristics on survival. The data was subsetted into core samples and leading edge samples, and were analysed separately by tumour morphology in the leading edge and core specimens, grading the intensity and percentage positivity in both the epithilium and stroma.

### 4.4. MTT Assay

To assess the cell viability, OE21 and OE33 cells were seeded in 96-well plates at a concentration of 1.0 × 10^4^ cells/mL for 24 h and were allowed to adhere to the plate overnight. The cells were then drug treated using STATTIC 50, 100, and 300 μM, or an RPMI medium control, for 24 h. An MTT reagent that was dissolved in the RPMI media was added to the wells to a final concentration of 0.5 mg/mL and was incubated for 2 h at 37 °C. The media was removed, the cells were washed with PBS and 100 μL of DMSO added to each well to lyse the cells. The 96-well plate was incubated in the dark for 5 min to allow colour development and was read immediately at 450 nm, using the Versa Max microplate reader (Molecular Devices, Sunnyvale, CA, USA) to determine a viable cell number. All of the data were analysed from three independent experiments.

### 4.5. BrdU Proliferation Assay

For the assessment of the cell proliferation, the cells were seeded at a dilution of 2.5 × 10^4^/well in 96-well plates in the appropriate complete media, and were allowed to adhere overnight at 37 °C. Following this, they were incubated overnight in serum-depleted media (0.5% FBS) and were treated with STATTIC, 100 and 300 μM, for 24 h or the RPMI medium control. The cell proliferation was then assessed using a BrdU cell proliferation ELISA (Roche Diagnostics Ltd., Sussex, UK) according to the manufacturer’s guidelines. Absorbance was measured on an Alpha Flour Plus plate reader (Tecan Trading AG, Männedorf, Switzerland) at 450 nm, with the reference set to 690 nm. Wells containing cells but no BrdU label were used to subtract the background absorbances and the percentage increase/decrease in proliferation was calculated relative to the untreated cells. All of the data were analysed from three independent experiments.

### 4.6. xCelligence Migration Assay 

The Roche xCelligence system was utilized for the assessment of cell migration. The RTCA DP instrument used a 16-well cellular invasion/migration plate with microelectric sensors integrated onto the underside of a microporous polyethylene terephthalate membrane of a Boyden-like chamber. The quantitative determination of the migrated cells was determined via a cell-index value, which was reflective of the impedance change on the underside of the membrane resultant from cell migration. Therefore, the cell migration activity could be determined in real time via the cell index profile. For the assessment of the cell migration, 160 μL of the RPMI medium with chemoattractant (10% FBS) at 37 °C was placed in the lower chamber, forming a meniscus. The upper chamber was then applied and 30 μL of a serum-free medium placed within each well. This was placed within the cradle of the RTCA system and the experimental parameters were determined. The duration of recording was set to 48 h, with a recording sweep set at 10 min intervals. The media-only wells were used to obtain a baseline impedance measurement. At 24 h prior to the experiment, the cells were seeded at 180,000 cells per well, in 60 × 15 mm cell culture plates, and were allowed to adhere in the RPMI 1640 medium with 10% Fetal Bovine Serum (FBS) and 1% Penicillin/Streptomycin. The cells were washed with PBS and trypsinsed, as previously described. The cells were resuspended in 1 mL of serum-free medium. The cells were counted, as previously described, and were further diluted using a serum-free treatment, such that a 100 μL volume of cell suspension containing 40,000 viable cells was added to the upper chamber of the xCelligence plate, and the experiment was commenced. Treatment with STATTIC 300 μM or the RPMI medium were performed in duplicate, and each experiment was performed in three independent experiments.

### 4.7. Annexin-V-FITC/Propidium Iodide Apoptosis Assay

Apoptosis was measured using annexin V-FITC and propidium iodide staining and was assessed by flow cytometry. The cells were seeded at a density of 3 × 10^4^ cells in 96-well plates and were allowed to adhere overnight at 37 °C in 5% CO_2_/95% humidified air. The cells were cultured in STATTIC, 100 and 300 μM, or the RPMI medium control for 24 h. The cells were then trypsinised and resuspended 1× stock binding buffer (binding buffer 10×: 0.1 M HEPES, pH 7.4; 1.4 M NaCl; 25 mM CaCl2). Then, 3 μL of Annexin V-FITC (IQ products, Groningen, The Netherlands) was added to each sample, apart from the controls, and was incubated at 4 °C for 15–20 min in the dark. Following the resuspension in the 150 μL binding buffer, 150 μL of 1:4000 PI (Invitrogen, Carlsbad, CA, USA) was added to the appropriate samples and the apoptosis was measured using a CyAN ADP (Beckman Coulter, Brea, CA, USA) flow cytometer. The data were analysed using FlowJo X 10.07r2 software (TreeStar, Inc., Ashland, Oregon). The percentage of apoptosis in the treated cells was expressed relative to the untreated control cells from three independent experiments.

### 4.8. Statistical Analysis

#### 4.8.1. In Vitro

The normality of the data were assessed and the non-parametric tests were used where appropriate. Continuous variables were compared using unpaired *t*-tests for the normally distributed data, and Mann–Whitney U test was used otherwise. A significance level of 0.05 was used for all of the analyses and all of the *p* values that were reported were two-tailed. The statistical analysis was performed using Graphpad Prism version 6 for Mac OS X (Graphpad Prism Software, Inc. San Diego, California, USA).

#### 4.8.2. Ex Vivo

The Cox Proportional Hazard regression was used to assess the influence of the individual biomarkers and tumour characteristics on survival. The proportional hazards assumption was verified by inspection of the Kaplan–Meier curves and Schoenfeld residual plots, and the time-varying effects were added where appropriate. The analysis was done with the full-range continuous pSTAT3 level and was split into quartiles for visualization purposes (Supplemental Figures S1 and S2). For the TMA analysis, the data were divided into core samples and leading edge samples, and were analysed separately by tumour morphology. Categorical predictor variables with a low frequency were dropped unless at least two levels of the predictor had at least five observations. The hazard ratios and confidence intervals were reported. A significance level of 0.05 was used for all of the analyses and all of the *p* values that were reported were two-tailed. The statistical analysis was performed using SPSSversion 20.0 software (SPSS, Chicago, IL, USA) and Graphpad Prism version 6 for Mac OS X (Graphpad Prism Software, Inc.).

## 5. Conclusions

We have shown in this study that OAC and SCC exhibit divergent STAT3 expression patterns. There are increased levels of IL-6R in SCC tumours, relative to OAC, yet a correlation between the IL-6R levels and pSTAT3 is detected in both of the histological subtypes. Importantly, despite similar levels of pSTAT3 in both OAC and SCC, it is associated with adverse survival in SCC, but it has a protective effect in OAC. The STAT3 inhibition resulted in the important functional effects of reduced viability, proliferation, migration, and an increase in apoptosis in both of the histological subtypes. STAT3 is an interesting therapeutic target in this context, and a further understanding of the reasons for divergent STAT3 signaling and the resultant cellular effects will be important for the development of therapeutic targets in each cancer subtype.

## Figures and Tables

**Table 1:** The Cox regression analysis of association of the pSTAT3 and mortality in oesophageal adenocarcinoma and squamous cell carcinoma. The percentage positivity in both the epithelium and stroma are shown, and the intensity for the tissue as a whole is also shown.

	Core			Leading Edge		
Adenocarcinoma						
	HR	95% CI	*p*-value	HR	95% CI	*p*-value
Intensity	1.107	0.661, 1.856	0.699	0.449	0.194, 1.037	0.061
Epithelium	1.065	0.843, 1.345	0.600	0.740	0.574, 0.953	0.020
Stroma	1.192	0.890, 1.598	0.239	0.660	0.425, 1.025	0.064
Squamous Cell Carcinoma						
Intensity	1.164	0.288, 4.701	0.831	1.514	0.327, 7.015	0.596
Epithelium	1.189	0.768, 1.839	0.437	3.726	0.871, 15.938	0.076
Stroma	6.382	1.266, 32.184	0.025	1.222	0.419, 3.570	0.713

**Table 2:** Demographic details of study population.

		Adenocarcinoma (*n* = 116)	Squamous Cell Carcinoma (*n* = 38)	Total (*n* = 154)
Age	Mean ± sd	64.53 ± 11.75	63.63 ± 11.4	64.48 ± 11.18
BMI	Mean ± sd	26.3 ± 4.5 (88)	23.4 ± 4.2 (30)	25.6 ± 4.6 (118)
Involvement *n* (%)				
Lymph node		79 (69.3)	21 (56.8)	100 (66.2)
Venous		55 (49.5)	15 (40.5)	70 (47.3)
Perineural		50 (43.9)	8 (21.6)	58 (38.4)
Involved resection margins		62 (54.4)	25 (65.8)	87 (57.2)
Differentiation				
	Well	6 (5.2)	5 (13.2)	11 (7.1)
	Moderate	67 (57.8)	24 (63.2)	91 (59.1)
	Poor	43 (37.1)	9 (23.7)	52 (33.8)
Microscopic residual tumour		29 (25.9)	8 (21.6)	37 (24.8)
Staging *n* (%)				
T Stage	In situ	0	1 (2.7)	1 (0.7)
	T1	14 (12.2)	3 (8.1)	17 (11.2)
	T2	20 (17.4)	7 (18.9)	27 (17.8)
	T3	77 (67.0)	25 (67.6)	102 (67.1)
	T4	4 (3.5)	1 (2.7)	5 (3.3)
N stage	N0	39 (33.9)	14 (37.8)	53 (34.9)
	N1	62 (53.9)	23 (62.2)	85 (55.9)
	N2	9 (7.8)	0	9 (5.9)
	N3	5 (4.3)	0	5 (3.3)
M stage	M1	3 (18.8)	2 (40.0)	5 (23.8)

## Supplementary Materials

Supplementary materials can be found at http://www.mdpi.com/1422-0067/19/6/1720/s1.

## References

[1] Chavan S., Bray F., Lortet-Tieulent J., Goodman M., Jemal A. (2014). International variations in bladder cancer incidence and mortality. Eur. Urol..

[2] Siegel R., Naishadham D., Jemal A. (2013). Cancer statistics, 2013. CA Cancer J. Clin..

[3] Torjesen I. (2014). Large personalised medicine trial in lung cancer heralds new research partnership. BMJ.

[4] Judd L.M., Menheniott T.R., Ling H., Jackson C.B., Howlett M., Kalantzis A., Priebe W., Giraud A.S. (2014). Inhibition of the JAK2/STAT3 pathway reduces gastric cancer growth in vitro and in vivo. PLoS ONE.

[5] Darnell J.E., Kerr I.M., Stark G.R. (1994). Jak-STAT pathways and transcriptional activation in response to IFNs and other extracellular signaling proteins. Science.

[6] White C.A., Nicola N.A. (2013). SOCS3: An essential physiological inhibitor of signaling by interleukin-6 and G-CSF family cytokines. JAKSTAT.

[7] Bromberg J.F., Wrzeszczynska M.H., Devgan G., Zhao Y., Pestell R.G., Albanese C., Darnell J.E. (1999). Stat3 as an oncogene. Cell.

[8] Lapeire L., Hendrix A., Lambein K., Van Bockstal M., Braems G., Van Den Broecke R., Limame R., Mestdagh P., Vandesompele J., Vanhove C. (2014). Cancer-associated adipose tissue promotes breast cancer progression by paracrine oncostatin M and Jak/STAT3 signaling. Cancer Res..

[9] Hodge D.R., Hurt E.M., Farrar W.L. (2005). The role of IL-6 and STAT3 in inflammation and cancer. Eur. J. Cancer.

[10] Pai R., Lin C., Tran T., Tarnawski A. (2005). Leptin activates STAT and ERK2 pathways and induces gastric cancer cell proliferation. Biochem. Biophys. Res. Commun..

[11] Huo Y.Q., Ruan X., DU X., Shang L., Cai Y., Xu X., Wang M.R., Zhang Y., Fu S.B. (2013). Overexpression of p-Stat3 and Mcl-1, and their correlation with differentiation and apoptotic resistance in esophageal squamous cell carcinoma. Zhonghua Zhong Liu Za Zhi.

[12] You Z., Xu D., Ji J., Guo W., Zhu W., He J. (2012). JAK/STAT signal pathway activation promotes progression and survival of human oesophageal squamous cell carcinoma. Clin. Transl. Oncol..

[13] Wang X.H., Li S.S., Yan A.H., Lu C.X., Guo Y.P. (2006). Constitutive activation of signal transducers and activators of transcription 3 and expression of its target gene products in human ESCC cell line. Nan Fang Yi Ke Da Xue Xue Bao.

[14] Renehan A.G., Tyson M., Egger M., Heller R.F., Zwahlen M. (2008). Body-mass index and incidence of cancer: A systematic review and meta-analysis of prospective observational studies. Lancet.

[15] Timme S., Ihde S., Fichter C.D., Waehle V., Bogatyreva L., Atanasov K., Kohler I., Schöpflin A., Geddert H., Faller G. (2014). STAT3 expression, activity and functional consequences of STAT3 inhibition in esophageal squamous cell carcinomas and Barrett’s adenocarcinomas. Oncogene.

[16] Schust J., Sperl B., Hollis A., Mayer T.U., Berg T. (2006). Stattic: A small-molecule inhibitor of STAT3 activation and dimerization. Chem. Biol..

[17] Sellier H., Rébillard A., Guette C., Barré B., Coqueret O. (2013). How should we define STAT3 as an oncogene and as a potential target for therapy?. JAKSTAT,.

[18] Suh Y.A., Jo S.Y., Lee H.Y., Lee C. (2015). Inhibition of IL-6/STAT3 axis and targeting Axl and Tyro3 receptor tyrosine kinases by apigenin circumvent taxol resistance in ovarian cancer cells. Int. J. Oncol..

[19] Fofaria N.M., Srivastava S.K. (2015). STAT3 induces anoikis resistance, promotes cell invasion and metastatic potential in pancreatic cancer cells. Carcinogenesis.

[20] Levidou G., Sachanas S., Pangalis G., Kalpadakis C., Yiakoumis X., Moschogiannis M., Kyrtsonis M.C., Vassilakopoulos T., Tsirkinidis P., Kontopidou F. (2014). Immunohistochemical analysis of IL-6, IL-8/CXCR2 axis, Tyr p-STAT-3, and SOCS-3 in lymph nodes from patients with chronic lymphocytic leukemia: Correlation between microvascular characteristics and prognostic significance. Biomed. Res. Int..

[21] Bi X., Huang Z., Zhao J., Han Y., Li Z., Zhang Y., Li Y., Chen X., Hu X., Zhao H. (2015). Prognostic role of phosphor-STAT3 in patients with cancers of the digestive system: A systematic review and meta-analysis. PLoS ONE.

[22] Chen X., Ying Z., Lin X., Lin H., Wu J., Li M., Song L. (2013). Acylglycerol kinase augments JAK2/STAT3 signaling in esphageal squamous cells. J. Clin. Investig..

[23] Li H., Xiao W., Ma J., Zhang Y., Li R., Ye J., Wang X., Zhong X., Wang S. (2014). Dual high expression of STAT3 and cyclinD1 is associated with poor prognosis after curative resection of esophageal squamous cell carcinoma. Int. J. Clin. Exp. Pathol..

[24] Mesteri I., Schoppmann S.F., Preusser M., Birner P. (2014). Overexpression of CMET is associated with signal transducer and activator of transcription 3 activation and diminished prognosis in oesophageal adenocarcinoma but not in squamous cell carcinoma. Eur. J. Cancer.

[25] Yu H., Lee H., Herrmann A., Buettner R., Jove R. (2014). Revisiting STAT3 signalling in cancer: New and unexpected biological functions. Nat. Rev. Cancer.

[26] Zhang Q., Zhang C., He J., Guo Q., Hu D., Yang X., Wang J., Kang Y., She R., Wang Z. (2014). STAT3 inhibitor stattic enhances radiosensitivity in esophageal squamous cell carcinoma. Tumour Biol..

[27] Liu J.R., Wu W.J., Liu S.X., Zuo L.F., Wang Y., Yang J.Z., Nan Y.M. (2015). Nimesulide inhibits the growth of human esophageal carcinoma cells by inactivating the JAK2/STAT3 pathway. Pathol. Res. Pract..

[28] Schoppmann S.F., Jesch B., Friedrich J., Jomrich G., Maroske F., Birner P. (2012). Phosphorylation of signal transducer and activator of transcription 3 (STAT3) correlates with Her-2 status, carbonic anhydrase 9 expression and prognosis in esophageal cancer. Clin. Exp. Metast..

[29] Chen M.F., Chen P.T., Lu M.S., Lin P.Y., Chen W.C., Lee K.D. (2013). IL-6 expression predicts treatment response and outcome in squamous cell carcinoma of the esophagus. Mol. Cancer.

[30] Cao Y.Y., Yu J., Liu T.T., Yang K.X., Yang L.Y., Chen Q., Shi F., Hao J.J., Cai Y., Wang M.R. (2018). Plumbagin inhibits the proliferation and survival of esophageal cancer cells by blocking STAT3-PLK1-AKT signaling. Cell Death Dis..

[31] Gao S., Li S., Duan X., Gu Z., Ma Z., Yuan X., Feng X., Wang H. (2017). Inhibition of glycogen synthase kinase 3 beta (GSK3beta) suppresses the progression of esophageal squamous cell carcinoma by modifying STAT3 activity. Mol. Carcinog..

[32] Gu Z.F., Zhang Z.T., Wang J.Y., Xu B.B. (2017). Icariin exerts inhibitory effects on the growth and metastasis of KYSE70 human esophageal carcinoma cells via PI3K/AKT and STAT3 pathways. Environ. Toxicol. Pharmacol..

[33] Xuan X., Li S., Lou X., Zheng X., Li Y., Wang F., Gao Y., Zhang H., He H., Zeng Q. (2015). Stat3 promotes invasion of esophageal squamous cell carcinoma through up-regulation of MMP2. Mol. Biol. Rep..

[34] Rice T.W., Blackstone E.H., Rusch V.W. (2010). 7th edition of the AJCC Cancer Staging Manual: Esophagus and esophagogastric junction. Ann. Surg. Oncol..

