# Supplementary material for: pSTAT3 Levels Have Divergent Expression Patterns and Associations with Survival in Squamous Cell Carcinoma and Adenocarcinoma of the Oesophagus
Source: Int J Mol Sci. 2018 Jun 10;19(6):1720. doi: 10.3390/ijms19061720 (PMC6032321; doi:10.3390/ijms19061720)
Supplement: Supplementary file 1 [file ijms-19-01720-s001.pdf]

Supplementary Material

Supplementary Table 1. Associations of IL-6R staining with survival in OAC and SCC

	P Value
**Oesophageal Adenocarcinoma Leading Edge**	
Percentage Positivity Epithelium	0.78
Percentage Positivity Stroma	0.75
Percentage Positivity Epithelium x Stroma	0.83
Intensity	0.24
**Oesophageal Adenocarcinoma Core**	
Percentage Positivity Epithelium	0.48
Percentage Positivity Stroma	0.007
Percentage Positivity Epithelium x Stroma	0.46
Intensity	0.97
**Squamous Cell Carcinoma Leading Edge**	
Percentage Positivity Epithelium	0.46
Percentage Positivity Stroma	0.52
Percentage Positivity Epithelium x Stroma	0.89
Intensity	0.86
**Squamous Cell Carcinoma Core**	
Percentage Positivity Epithelium	0.57
Percentage Positivity Stroma	0.50
Percentage Positivity Epithelium x Stroma	0.39
Intensity	0.41

Differences of p<0.05 (*), p<0.01 (**) and p<0.001 (***) were considered statistically significant.

Supplementary Table 2. Correlation analysis of tumour differentiation, ratio of positive nodes, pathological T and N stage with IL-6R grading in leading edge and core OAC and SCC samples.

**Parameters compared**	**Adenocarcinoma**				**Squamous Cell Carcinoma**			
	Leading Edge		Core		Leading Edge		Core	
**Tumour Differentiation**	p-value	R value	p-value	R value	p-value	R value	p-value	R value
Intensity	0.49	0.08	0.34	0.096	0.28	-0.083	0.74	-0.235
% Positivity Epithelium	0.56	0.066	0.86	0.017	0.69	-0.065	0.79	-0.088
% Positivity Stroma	0.79	0.03	0.30	0.104	0.55	-0.14	0.58	-0.131
**Ratio of Positive Nodes**								
Intensity	0.28	-0.122	0.18	0.136	0.62	0.129	0.60	0.108

% Positivity Epithelium	0.53	-0.071	0.98	0.002	0.76	0.302	0.23	0.067
% Positivity Stroma	0.74	-0.037	0.70	0.039	0.14	0.348	0.15	0.312
**Pathological T stage**								
Intensity	0.41	0.093	0.80	0.026	0.83	-0.268	0.28	-0.047
% Positivity Epithelium	0.61	-0.057	0.99	0.04	0.43	-0.041	0.87	-0.172
% Positivity Stroma	0.67	-0.048	0.98	-0.002	0.72	-0.16	0.52	-0.077
**Pathological N Stage**								
Intensity	0.54	0.07	0.056	0.193	0.67	-0.093	0.71	0.092
% Positivity Epithelium	0.56	-0.065	0.66	0.044	0.79	0.035	0.89	-0.058
% Positivity Stroma	0.80	-0.028	0.51	0.065	0.45	0.076	0.76	-0.164

Results shown are p values from correlation analyses, Differences of p<0.05 (*), p<0.01 (**) and p<0.001 (***) were considered statistically significant.

Supplementary Table 3. Correlation analysis of tumour differentiation, ratio of positive nodes, pathological T and N stage with pSTAT3 grading in leading edge and core OAC and SCC samples

**Parameters compared**	**Adenocarcinoma**				**Squamous Cell Carcinoma**			
	Leading Edge		Core		Leading Edge		Core	
**Tumour Differentiation**	p-value	R value	p-value	R value	p-value	R value	p-value	R value
Intensity	0.89	-0.017	0.73	0.035	0.44	-0.191	0.42	-0.153
% Positivity Epithelium	0.56	-0.08	0.79	0.029	0.92	-0.023	0.80	-0.046
% Positivity Stroma	0.79	0.038	0.23	0.130	0.33	0.23	0.31	-0.199
**Ratio of Positive Nodes**								
Intensity	0.95	-0.007	0.50	-0.07	0.15	-0.347	0.16	-0.267
% Positivity Epithelium	0.63	-0.066	0.06	-0.205	0.37	-0.209	0.10	-0.306
% Positivity Stroma	0.20	0.186	0.54	-0.065	0.76	0.073	0.06	-0.362
**Pathological T stage**								
Intensity	0.93	0.01	0.87	-0.017	0.45	-0.187	0.23	-0.227
% Positivity Epithelium	0.31	0.142	0.68	0.045	0.64	-0.109	0.97	0.005

% Positivity Stroma	0.48	0.488	0.73	0.036	0.58	-0.129	0.81	0.047
**Pathological N Stage**								
Intensity	0.96	0.006	0.63	-0.05	0.85	0.046	0.36	-0.175
% Positivity Epithelium	0.93	0.012	**0.05**	-0.214	0.98	0.003	0.62	0.096
% Positivity Stroma	0.13	0.217	0.81	-0.026	0.85	0.043	0.25	-0.224

Results shown are p values from correlation analyses, Differences of p<0.05 (*), p<0.01 (**) and p<0.001 (***) were considered statistically significant.

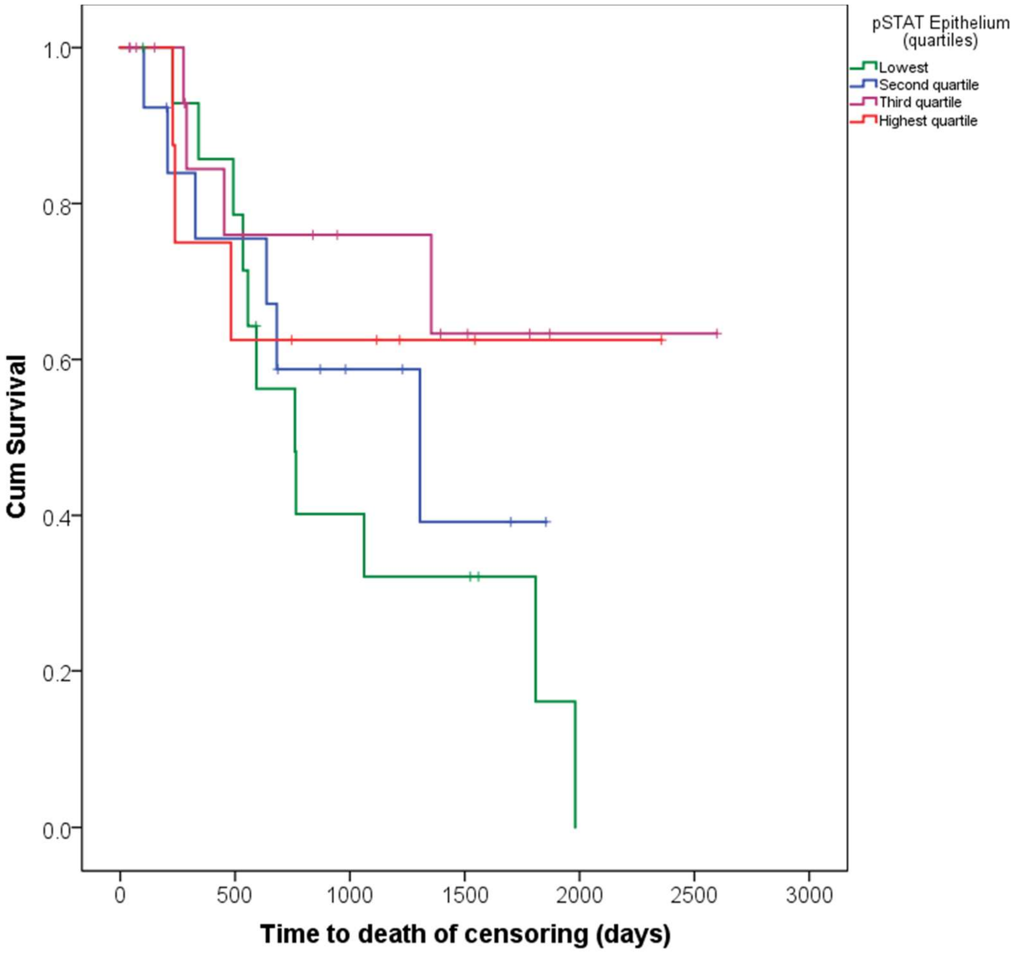

Supplemental Figure 1. Kaplan Meier curves of the association between pSTAT3 percentage positivity in epithelium and mortality in leading edge OAC samples. Data is represented in quartiles. (Quartile 1; ≤3.5, quartile 2; >3.5 and ≤ 4.5, quartile 3; >4.5 and ≤ 5, quartile 4; >5). Differences of p<0.05 (*), p<0.01 (**) and p<0.001 (***) were considered statistically significant.

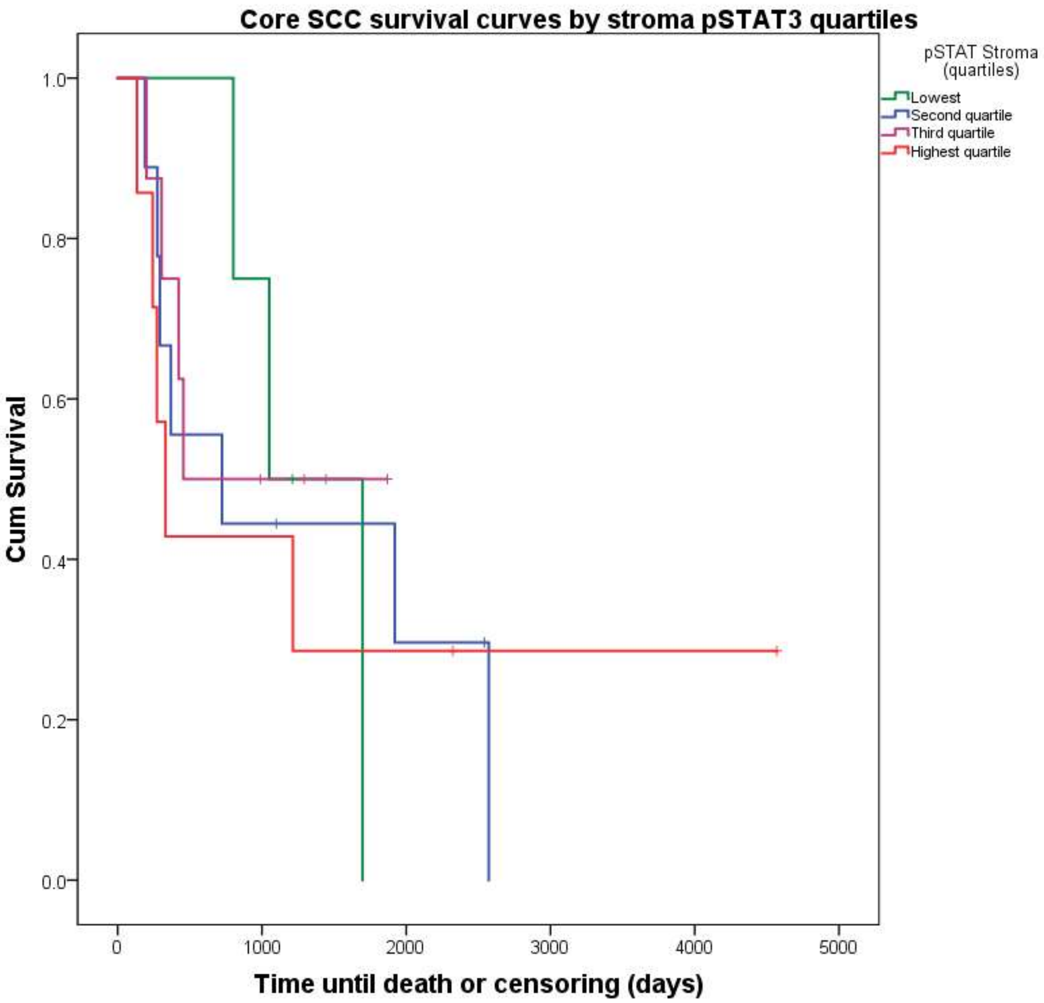

Supplemental Figure 2. Kaplan Meier curves of the association between pSTAT3 percentage positivity in stroma and mortality in core SCC samples. Data is represented in quartiles. (Quartile 1; ≤3.66, quartile 2; >3.66 and ≤ 4, quartile 3; >4 and ≤ 4.66, quartile 4; >4.66). Differences of p<0.05 (*), p<0.01 (**) and p<0.001 (***) were considered statistically significant.

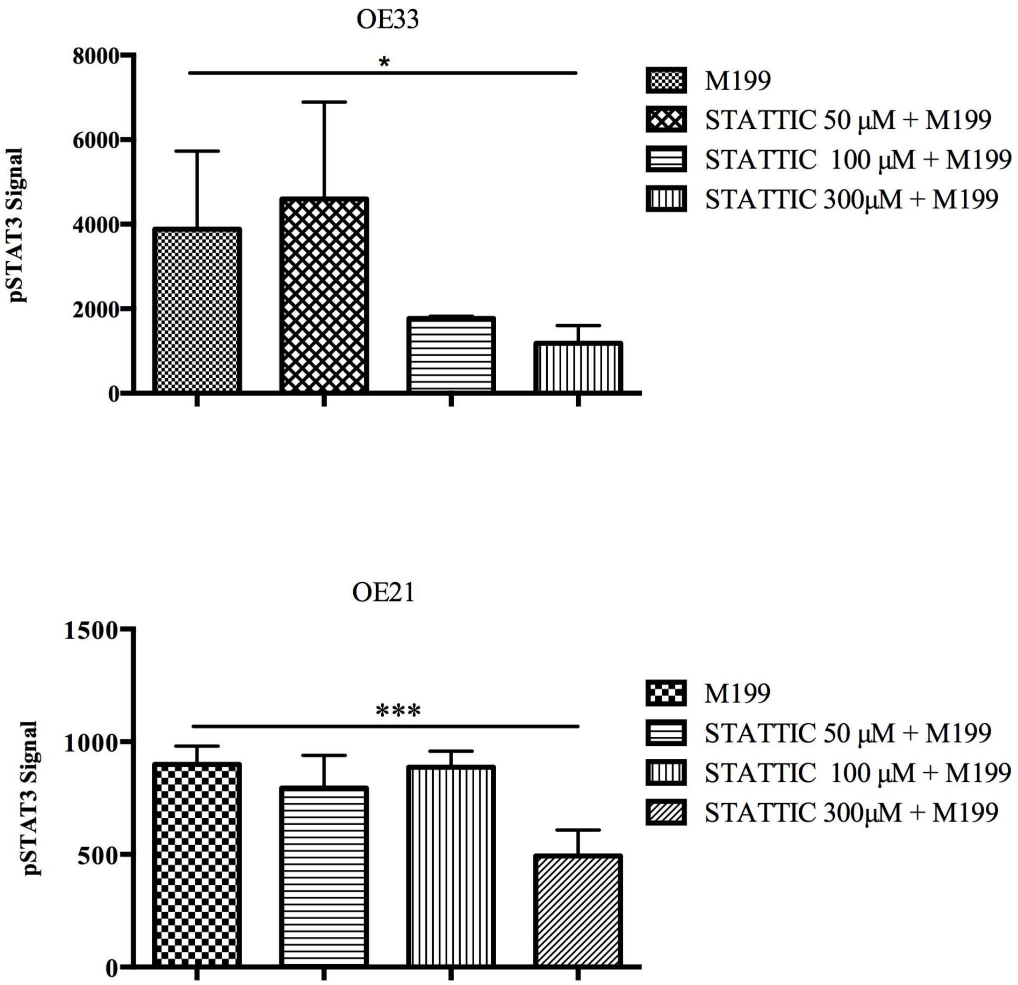

Supplementary Figure 3. The effect of pSTAT3 inhibitor STATTIC on pSTAT3 levels in OE33 and OE21 cells. Results are compared to controls treated with M199 medium alone. N=3. Bars denote mean ± standard deviation. P value was estimated using student's t-test. Differences of $p < 0.05$ (*), $p < 0.01$ (**) and $p < 0.001$ (***) were considered statistically significant.
